# Genome-wide methylation analysis identifies a core set of hypermethylated genes in CIMP-H colorectal cancer

**DOI:** 10.1186/s12885-017-3226-4

**Published:** 2017-03-28

**Authors:** Tyler McInnes, Donghui Zou, Dasari S. Rao, Francesca M. Munro, Vicky L. Phillips, John L. McCall, Michael A. Black, Anthony E. Reeve, Parry J. Guilford

**Affiliations:** 1 Cancer Genetics Laboratory, Centre for Translational Cancer Research (Te Aho Matatū), Department of Biochemistry, University of Otago, Dunedin, 9054 New Zealand; 2 Department of Surgical Sciences, Dunedin School of Medicine, University of Otago, Dunedin, 9054 New Zealand

**Keywords:** Epigenome, Methylation, Colorectal cancer, CIMP

## Abstract

**Background:**

Aberrant DNA methylation profiles are a characteristic of all known cancer types, epitomized by the CpG island methylator phenotype (CIMP) in colorectal cancer (CRC). Hypermethylation has been observed at CpG islands throughout the genome, but it is unclear which factors determine whether an individual island becomes methylated in cancer.

**Methods:**

DNA methylation in CRC was analysed using the Illumina HumanMethylation450K array. Differentially methylated loci were identified using Significance Analysis of Microarrays (SAM) and the Wilcoxon Signed Rank (WSR) test. Unsupervised hierarchical clustering was used to identify methylation subtypes in CRC.

**Results:**

In this study we characterized the DNA methylation profiles of 94 CRC tissues and their matched normal counterparts. Consistent with previous studies, unsupervized hierarchical clustering of genome-wide methylation data identified three subtypes within the tumour samples, designated CIMP-H, CIMP-L and CIMP-N, that showed high, low and very low methylation levels, respectively. Differential methylation between normal and tumour samples was analysed at the individual CpG level, and at the gene level. The distribution of hypermethylation in CIMP-N tumours showed high inter-tumour variability and appeared to be highly stochastic in nature, whereas CIMP-H tumours exhibited consistent hypermethylation at a subset of genes, in addition to a highly variable background of hypermethylated genes. *EYA4, TFPI2* and *TLX1* were hypermethylated in more than 90% of all tumours examined. One-hundred thirty-two genes were hypermethylated in 100% of CIMP-H tumours studied and these were highly enriched for functions relating to skeletal system development (Bonferroni adjusted *p* value =2.88E-15), segment specification (adjusted *p* value =9.62E-11), embryonic development (adjusted *p* value =1.52E-04), mesoderm development (adjusted *p* value =1.14E-20), and ectoderm development (adjusted *p* value =7.94E-16).

**Conclusions:**

Our genome-wide characterization of DNA methylation in colorectal cancer has identified 132 genes hypermethylated in 100% of CIMP-H samples. Three genes, *EYA4*, *TLX1* and *TFPI2* are hypermethylated in >90% of all tumour samples, regardless of CIMP subtype.

**Electronic supplementary material:**

The online version of this article (doi:10.1186/s12885-017-3226-4) contains supplementary material, which is available to authorized users.

## Background

Colorectal cancer (CRC) is a prevalent disease, particularly in the Western world, with 1.36 mm cases diagnosed worldwide in 2012 [1]. As with all cancers, CRC encompasses multiple molecular subtypes with specific characteristics [2]. The CpG island methylator phenotype (CIMP) is one subtype, and describes tumours with a high frequency of hypermethylation at CpG islands [3].

While there is no consensus on a gene panel to determine the CIMP status of a tumour, one of the most commonly used is the Weisenberger panel of genes comprising of *CACNA1G, NEUROG1, RUNX3, SOCS1* and *IGF2* [4]. CIMP can be further split into CIMP-high (CIMP-H) and CIMP-low (CIMP-L), which show high and intermediate levels of hypermethylation respectively [5]. The CIMP-L subtype, defined as tumours with 1/5 to 3/5 of these marker genes methylated, is associated with *KRAS* mutations and is more common in men [5]. CIMP-H tumours, defined as tumours with hypermethylation at >3/5 marker genes, are significantly associated with mutations in *BRAF*, female patients and location in the proximal colon [4, 5]. Recently, colorectal tumours have been split into further methylation subtypes. Hinoue et al. identified four subtypes based on hierarchical clustering of DNA methylation at loci exhibiting high inter-tumour variability [6]. Two, representing CIMP-H and CIMP-L tumours, were associated with *BRAF* and *KRAS* mutations, respectively. Tumours in the third cluster were associated with *TP53* mutations and prevalence in the distal colon, while the fourth cluster was enriched for tumours from the rectum, with low rates of *KRAS* and *TP53* mutations.

Hypermethylation occurs primarily at CpG islands, the majority of which are unmethylated in normal tissue and are found near the promoter region of approximately 70% of mammalian genes. ChIP-Seq experiments have demonstrated proteins including KDM2A and CFP1 preferentially bind unmethylated CpG islands [7, 8]. The regions surrounding CpG islands, termed island shores, are important for cellular differentiation and are also targets of aberrant methylation in cancer [9]. Hypermethylation in cancer occurs preferentially at genes that, in embryonic stem cells, exhibit the repressive H3K27me3 histone modification laid down by the Polycomb group (PcG) proteins [10]. Cells lacking members of the PcG complex are unable to complete normal cellular differentiation [11]. Many H3K27me3 marked genes also harbor the activating H3K4me3 mark in embryonic stem cells, a state referred to as ‘bivalent’, and these genes are enriched for roles in development and differentiation [12, 13]. Preferential hypermethylation of developmental and differentiation genes supports the epigenetic switching model, in which developmental regulators that are temporarily silenced by histone modification in stem or progenitor cells are often heavily DNA methylated in cancer [14]. This model proposes that bivalent genes, which would normally lose PcG protein occupancy and become upregulated, are maintained in a stably repressed state by the presence of aberrant DNA methylation, inhibiting differentiation [14, 15].

In this study, we characterized global cancer-specific methylation patterns of 94 CRC tumour samples and matched tissues at very high resolution. We find the frequency of hypermethylation at genes follows a steady continuum from CIMP-N to CIMP-L to CIMP-H tumours. We identified a core set of 132 genes that were hypermethylated in all CIMP-H tumours and associated preferentially with genes involved in development and differentiation.

## Methods

### Sample processing

Colorectal tumour samples and adjacent normal tissue (approximately 10 cm from the tumour) were obtained from Dunedin hospital, New Zealand. Samples were stored frozen and stored at −80 °C. DNA was extracted using the Quick-gDNA miniPrep kit (Zymo Research) and quantified using a NanoDrop. 1000 ng of DNA was bisulfite converted using the EZ DNA methylation kit (Zymo Research). Bisulfite conversion efficiency was measured using qRT-PCR and 100% methylated and 100% unmethylated DNA references with primers designed for ALU repeat regions, as described previously [16].

### Molecular characterisation

Microsatellite instability (MSI) was assessed using the mononucleotide repeat markers *BAT-26* and *NR-24* [17]. CIMP status was assessed using MethyLight [18] and the five-marker panel comprized of *CACNA1G, IGF2, NEUROG1, RUNX3* and *SOCS1* [4]. *KRAS* (G12 V) and *BRAF* (V600E) mutations were assessed using PCR and DNA sequencing. Primers were designed by [19] and obtained from IDT.

### Genome-wide methylation assay

Illumina Infinium HumanMethylation 450 K arrays were used to measure the ratio between the intensity of methylated and unmethylated alleles at 485577 CpG sites according to the manufacturer’s specifications. DNA methylation was scored as a β value (intensity of Methylated allele/(intensity of Methylated allele + intensity of Unmethylated allele +100) which ranges from 0 (fully unmethylated) to 1 (fully methylated) [20]. Probes not statistically significantly different from negative control probes (*p* value >0.05) were removed. Matched tissue pairs were processed on the same chip. Probes were rescaled for each sample so that internal control probes have a common mean across samples. CpGs located on the X or Y chromosomes, or known to cross-react with other regions of the genome, were removed [21]. Methylation at the remaining 371,377 CpGs was corrected for batch effects (between-array effects) using COMBAT [22].

### Statistical analysis

The software package MeV, version 4.9.0, was used to carry out Significance Analysis of Microarrays (SAM) and unsupervized hierarchical clustering using Euclidian distance and complete linkage [23]. SAM was used to identify differentially methylated CpGs by performing a non-parametric t-test for each probe on the array. SAM calculates the strength of the relationship between DNA methylation and the normal and tumour tissue groups followed by permutation testing to determine a False Discovery Rate (FDR). Statistical analysis and visualisation were carried out using the R/Bioconductor software packages [24]. *P* values were adjusted for multiple testing using the false-discovery rate (Benjamini-Hochberg) or Bonferroni method according to the specific R package and are referred to in the text.

The Wilcoxon signed rank test was used to identify differentially methylated regions between pairs of matched tumour-normal tissue samples. *P* values were adjusted for multiple testing using the false-discovery rate (Benjamini-Hochberg).

### Gene ontology

The online software tool PANTHER was used to identify biological processes enriched within genes associated with differentially methylated CpG islands [25]. The background gene list was the set of genes associated with the 12,600 CpG islands which were analysed for differential methylation. Gene ontology for individual CpG probes with differential methylation was carried out using the gometh function in the missMethyl package [26].

## Results

### Dataset

An Illumina Infinium 450 K methylation dataset was generated for 94 pairs of matched tumour/normal tissue. DNA methylation was interrogated at 485577 CpG sites located in CpG islands, island shores (<2 kb outside of the CpG island), island shelves (2 kb to 3 kb distant from the island), intergenic regions and gene bodies (GEO Accession No. GSE77718).

### Classification of CIMP status

**Fig. 1:**
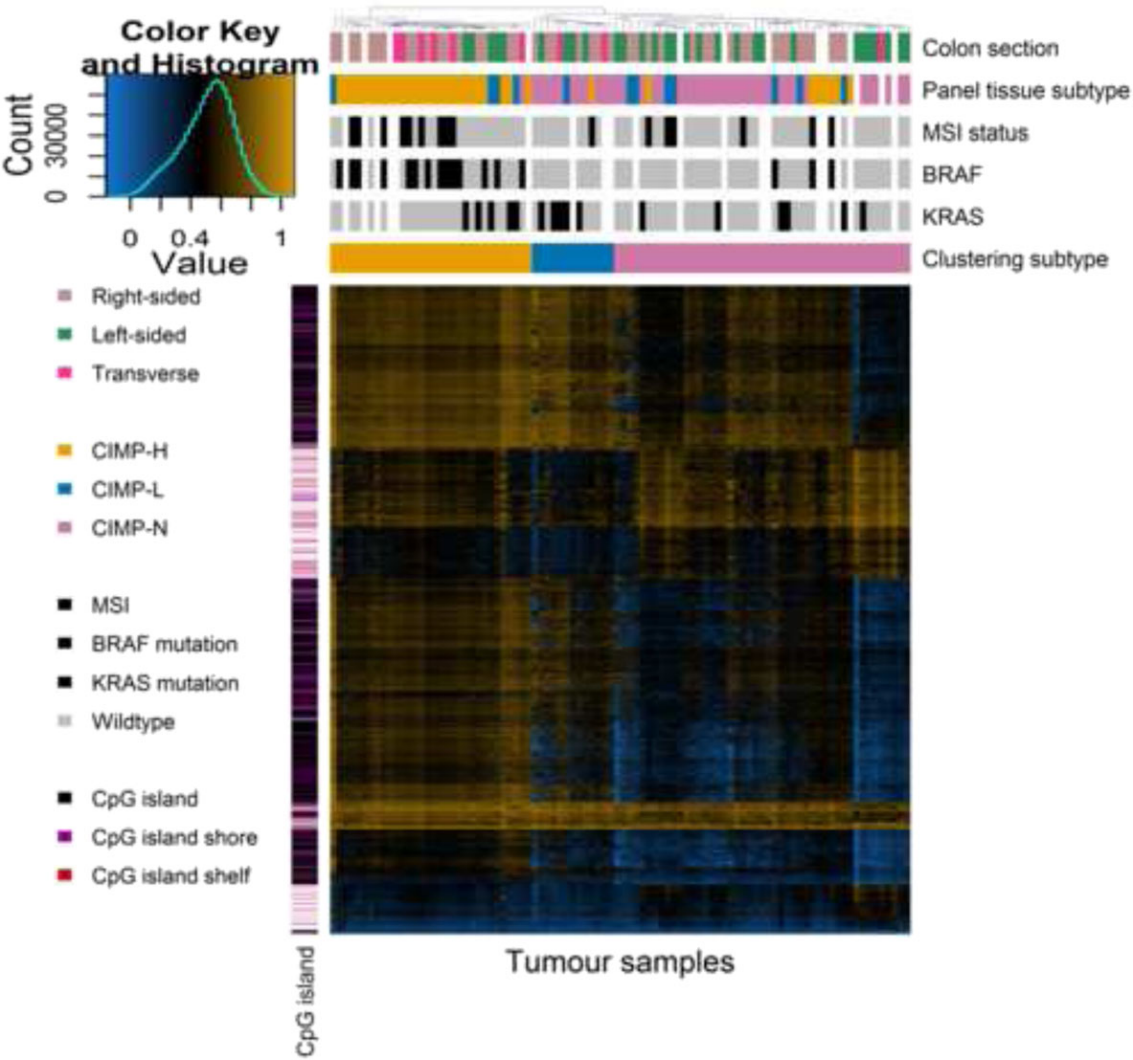
Heatmap. Representative section of a heatmap produced using unsupervized hierarchical clustering based on 20,573 differentially methylated probes. DNA methylation is represented by colour (*blue* indicates low methylation, *yellow* indicates high methylation). Clinical features (location of the tumour in the colon, CIMP status determined by the Weisenberger panel of genes, microstatellite instability (MSI) status, *BRAF* and *KRAS* mutation status, and hierarchical clustering status) of the tumour samples are indicated *above* the heatmap

To examine the differences in patterns of CpG methylation between tumours from different molecular subtypes we performed hierarchical clustering of all normal and tumour samples. Significance analysis of microarrays (SAM), which calculates a non-parametric t-test between tumour and normal tissue samples for each CpG on the array, was used to identify the 20,000 most differentially methylated CpGs. Hierarchical clustering of samples was then carried out using these 20,000 probes (Fig. 1). Only 2/94 normal samples clustered with tumour samples (omitted from further analysis), and 7/94 tumour samples clustered as normal tissue (we classified these tumour samples as CIMP-N tumours). The hierarchical clustering dendrogram supported three distinct tumour groups: a heavily methylated cluster designated CIMP-H (*n* = 32), a low methylation cluster designated CIMP-N (*n* = 47), and an intermediate cluster designated CIMP-L (*n* = 13). Clinical data was available for 26, 10 and 37 CIMP-H, CIMP-L and CIMP-N tumours respectively. Consistent with previous studies CIMP-H tumours were enriched (21/26, 81%, *p* value =0.016 using Welch *t* test) in the proximal colon and were associated with *BRAF* (V600E) mutations (14/26, 54%, *p* value <0.001). CIMP-N tumours were enriched (19/37, 51%, *p* value =0.016) in the distal colon and rectum [4, 6, 27] (Table 1).

**Table 1:** Clinical data

Mutations and MSI	CIMP-H (true *n* = 32, clinical *n* = 26)	CIMP-L (true *n* = 13, clinical *n* = 10)	CIMP-N (true *n* = 47, clinical *n* = 37)	*P* value
BRAF mutant	14 (44%, 54%)	0 (0%)	3 (6%, 8%)	< 0.001
BRAF wildtype	12 (38%, 46%)	10 (77%, 100%)	34 (72%, 92%)	
KRAS mutant	5 (16% 19%)	5 (38%, 50%)	6 (13%, 16%)	0.039^a^
KRAS wildtype	21 (66%, 81%)	5 (38%, 50%)	31 (66%, 84%)	
MSI	9 (28%, 35%)	1 (8%, 10%)	6 (13%, 16%)	0.133
MSS	17 (53%, 65%	9 (69%, 90%)	31 (66%, 84%)	
Colon location				
Proximal	21 (66%, 81%)	7 (54%, 70%)	18 (38%, 49%)	0.016
Distal	5 (16%, 19%)	3 (23%, 30%)	19 (40%, 51%)	
Differentiation				
Moderate	10 (31%, 38%)	9 (69%, 90%)	30 (64%, 81%)	0.012
Moderate, mucinous	7 (22%, 27%)	1 (8%, 10%)	5 (11%, 14%)	0.197
Poor	4 (13%, 15%)	0 (0%)	2 (4%, 5%)	0.209
Well	4 (13%, 15%)	0 (0%)	0 (0%)	0.075
Gene panel CIMP status				
CIMP-H	27 (84%)	1 (8%)	7 (15%)	< 0.001
CIMP-L	4 (13%)	1 (8%)	7 (15%)	
CIMP-N	1 (3%)	11 (85%)	30 (64%)	< 0.001
Age (SD)	73.6 (7.7)	69.2 (11)	69 (12.3)	0.07

Values are presented as mean (standard deviation) for continuous variables and count (percentage) for categorical variables. *P* values are comparisons between CIMP-H and CIMP-N tumour subtypes and are Fisher’s exact test (binary data) or Welch *t* test (continuous data).

^a^Enrichment of KRAS mutations is a comparison between CIMP-L and CIMP-N tumour subtypes

To compare the CIMP classifications derived from our clustering analysis to those derived from the Weisenberger panel of genes [4], we analysed the methylation status of our tumour samples using MethyLight [18] at each gene from this panel (*CACNA1G, NEUROG1, SOCS1, IGF2, RUNX3*). The concordance between our genome-wide cluster analysis and the five-gene panel approach was 84, 8, and 64% for CIMP-H, CIMP-L, and CIMP-N tumours, respectively (Table 1). Tumours classified as CIMP-L under the panel method were redistributed into both CIMP-H and CIMP-N hierarchical clustering subtypes. All further references to the CIMP status of our samples is based on our hierarchical clustering classification.

### CIMP-H tumours are hypermethylated at a core set of genes

To enable us to identify the complement of differentially methylated genes in individual tumours, for those CpG islands associated with genes we combined the probes from CpG islands and island shores and then compared the overall level of tumour/normal differential methylation for each gene using the Wilcoxon Signed Rank test. A CpG island was classified as differentially methylated if the Wilcoxon test returned a *p* value <0.005 following adjustment for multiple testing (False Discovery Rate (FDR)). An additional, independent test was applied to any CpG island that passed statistical significance under the Wilcoxon test. The mean Beta value (which ranges from 0 to 1 and indicates the percentage of methylated and alleles in the sample) was calculated for each gene (using all CpG island and island shore probes) in tumour and normal samples. A gene was classified as hypermethylated if the mean Beta value in the tumour was greater than the matched normal mean by 0.1 or more. A difference of 0.1 was selected as the most meaningful threshold, as this identified the greatest difference in the average number of hypermethylated CpG islands between CIMP H compared to CIMP N tumours. The final list of differentially methylated CpG islands were both statistically significant under the Wilcoxon Signed Rank test and had a difference in the mean beta value greater than 0.1.

**Fig. 2:**
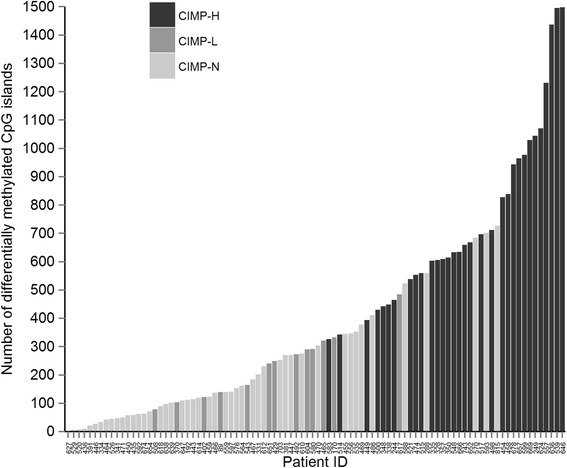
Inter-tumour variation in frequency of hypermethylated genes. The number of hypermethylated genes per tumour. *CIMP-N*, *CIMP-L* and *CIMP-H* tumour subtypes exhibit overlap in the number of hypermethylated genes

Individual tumours exhibited wide variation in the frequency of hypermethylated genes, ranging from 2 to 1498 hypermethylated genes per tumour (mean 397). The number of hypermethylated genes ranged from 2 to 727 (mean 196) in CIMP-N tumours, 78–485 in CIMP-L (mean 237) and 327–1498 in CIMP-H tumours (mean 759). Although the mean number of hypermethylation events in each CIMP group differed significantly, a continuum in hypermethylation frequency was observed with no clear boundary to divide the CIMP-H, CIMP-L and CIMP-N tumours (Fig. 2). The overlapping ranges of hypermethylated genes between the three tumour classes emphasizes that the classification of CIMP-H tumours is determined by both the number and the identity of hypermethylated genes.

**Fig. 3:**
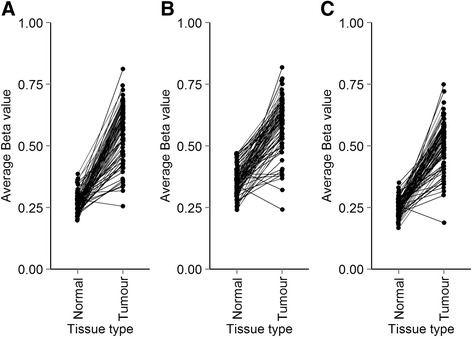
*EYA4*, *TFPI2* and *TLX1* are hypermethylated in more than 90% of all tumours analysed. The average Beta value from CpGs in the CpG island and island shore region of *EYA4* (**a**), *TFPI2* (**b**) and *TLX1* (**c**) for normal and tumour tissue samples

**Fig. 4:**
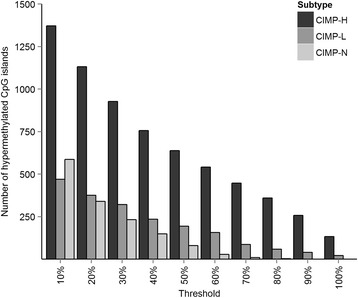
Frequency of consistently hypermethylated CpG islands. Tumours were divided into *CIMP groups*, and every CpG island was scored for the number of times it was methylated in tumours from each *CIMP group*. To visualize the number of frequently hypermethylated *CpG islands* a threshold between 10 and 100% was used, whereby an island would only be counted if it was hypermethylated in more than the specified percentage of tumours. The frequency (*Y axis*) is the number of CpG islands that are methylated in more than the percentage threshold indicated on the *X axis*

To determine the frequency of hypermethylation of individual genes in our tumour set we scored each gene for the number of times it was hypermethylated. Three genes (*EYA4, TFPI2,* and *TLX1*) were hypermethylated in more than 90% of patients, regardless of CIMP classification (Fig. 3). We identified 132 and 258 genes hypermethylated in 100 and 90% of CIMP-H tumours, respectively (Additional file 1: Table S1, Fig. 4). This included *EYA4*, *SFRP2* and *ALX4* which have been identified previously as hypermethylation targets [28–30]. Notably, our list of 132 genes often included multiple members of the same gene family. This included the Lim homeobox genes *LHX1/4/5/8/9*, members of the PAX family (*PAX1/2/3/5/6/9*) and the T-box transcription factor genes *TBX1/2/5/15*. PANTHER was used to identify functional enrichment within the 132 genes methylated in 100% of CIMP-H tumours (Table 2). The most enriched biological process was skeletal system development (Bonferroni adjusted *p* value =2.88E-15). Multiple developmental processes (segment specification, embryo, mesoderm and ectoderm development) were highly enriched (all terms with adjusted *p* value <1.52E-4). Terms relating to the regulation of transcription were also prevalent in the list of enriched biological processes. Gene ontology terms for the 258 genes hypermethylated in more than 90% of CIMP-H tumours also showed similar results.

**Table 2:** PANTHER gene ontology analysis of 132 genes hypermethylated in 100% of CIMP-H tumours

PANTHER GO-Slim Biological Process	Expected	Fold enrichment	*P* value
skeletal system development (GO:0001501)	1.4	14.8	2.88E-15
segment specification (GO:0007379)	1.1	13.9	9.62E-11
muscle organ development (GO:0007517)	1.8	12.7	2.35E-16
pattern specification process (GO:0007389)	1.4	11.4	3.08E-10
mesoderm development (GO:0007498)	3.2	10.1	1.14E-20
ectoderm development (GO:0007398)	3	9	7.94E-16
nervous system development (GO:0007399)	4.9	8.6	1.79E-25
embryo development (GO:0009790)	1.3	8	1.52E-04
system development (GO:0048731)	7	7.3	1.83E-28
heart development (GO:0007507)	1	7	1.71E-02
cellular defense response (GO:0006968)	1.1	6.5	2.78E-02
regulation of transcription from RNA polymerase II promoter (GO:0006357)	7.4	6.4	2.35E-23
transcription from RNA polymerase II promoter (GO:0006366)	9.3	5.2	3.85E-20
developmental process (GO:0032502)	13.2	5	3.25E-29
transcription, DNA-dependent (GO:0006351)	11.5	4.4	6.06E-18
cell differentiation (GO:0030154)	2.8	3.9	3.25E-02
RNA metabolic process (GO:0016070)	15.4	3.2	1.34E-12
nucleobase-containing compound metabolic process (GO:0006139)	23	2.2	1.11E-06
Unclassified (UNCLASSIFIED)	46.5	0.6	0.00E + 00

PANTHER was used to test for over represented gene ontology terms among the 132 genes associated with CpG islands hypermethylated in 100% of CIMP-H tumours. The background gene list was all genes which were tested for differential methylation

### Distribution of individual differentially methylated CpGs within the colorectal cancer genome

**Fig. 5:**
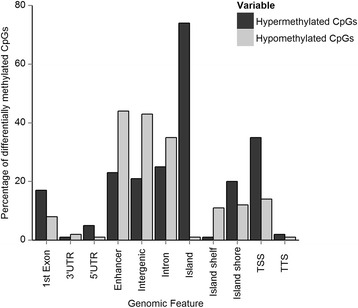
Genomic location of significantly differentially methylated CpGs. The percentage of hypermethylated and hypomethylated CpGs located in each genomic feature (1st exon, 3′ untranslated region (UTR), 5’UTR, enhancer, intergenic, intron, CpG island, CpG island shore, CpG island shelf, transcription start site (TSS, a window 1500 bp upstream of the TSS) and transcription termination site (TTS)). Probes may be located in more than one genomic feature e.g. CpG islands often overlap transcription start sites

The 20,000 most differentially methylated CpGs identified by SAM were annotated using the online probe software tool HOMER. At the CpG dinucleotide level, hypermethylation was more common, with 73% of the differentially methylated CpGs being hypermethylated in tumours. However, multiple probes were commonly found in the same gene, consequently there were similar numbers of hypermethylated (*n* = 3278) and hypomethylated genes (*n* = 3441). The majority (74%) of hypermethylated CpGs occurred in CpG islands rather than island shores (20%) or shelves (1%). This is consistent with previous studies finding 70–80% of CpGs hypermethylated in CIMP-H tumours were located within CpG islands [31, 32] but differs from a study using a custom-designed NimbleGen microarray which found cancer-associated DNA methylation occurred more frequently in the shores and shelves [9]. In contrast to the hypermethylated CpGs, we observed hypomethylated CpGs to be more frequently located in CpG island shore or shelf regions (12 and 11% of hypomethylated CpGs, respectively) compared to CpG islands themselves (1.3%) (Fig. 5).

Hypomethylated CpGs were most strongly enriched in enhancer regions (44%) and intergenic regions (43%). As the Illumina 450 K array contains more CpGs located in CpG islands than in CpG island shores or shelves, we also calculated the fraction of the total number of CpGs from each genomic feature that are found on the array. Ten percent of all CpG island probes exhibited differential methylation (either hyper- or hypomethylated), compared to 4% of island shore probes and 2% of island shelf probes, indicating CpG islands are the primary genomic feature of aberrant DNA methylation in CRC.

### Gene ontology for hyper- and hypo-methylated CpGs

Next we assessed the biological processes most commonly affected by changes in DNA methylation using the missMethyl R package [26]. We first interrogated groups of CpGs based on the genomic feature they were located in (for a list of genomic features refer to Fig. 5). Hypermethylation occurring within 1.5 kb of the transcription start site (TSS region) was enriched for nervous system development (*p* value =1.64E-20), neuron differentiation (*p* value =5.86E-16), multicellular organism development (*p* value 5.81E-15) and cell-cell signaling (*p* value =9.14E-15). Hypermethylation occurring in CpG islands was enriched for development (*p* value =3.28E-34), cell-cell signaling (*p* value =8.73E-30) and differentiation (*p* value =1.04E-28) related terms (all *p* values adjusted using FDR correction for multiple testing). CpG island shores, which have been implicated in tissue differentiation [9], were enriched for hypermethylation in multiple development related terms such as nervous system development (*p* value =9.58E-20) and anatomical structure development (*p* value =1.22E-13).

As a general trend our gene ontology analysis showed hypermethylation was more likely to be enriched for terms relating to differentiation, development, cell morphogenesis, homeobox genes, and DNA-binding. Hypomethylation was more commonly found at genes enriched for adhesion, cell signalling, plasma membrane parts and glycoproteins, consistent with earlier studies [6].

The above analyses identify individual CpGs with cancer-specific aberrant methylation and demonstrate these are useful in clustering CRC samples into subtypes.

## Discussion

In this study we utilized the high density coverage of the Illumina 450 K methylation array to characterize DNA methylation in CRC. Using this genome wide approach, and consistent with previous studies, we identified three methylation subtypes (high, intermediate, and low levels of methylation). The CIMP-H subtype was enriched for tumours from the proximal colon and female patients, as previously observed [4, 5]. Notably, only 84% of CIMP-H tumours in our dataset were classed as CIMP-H using the Weisenberger panel [4]. Using the Illumina Infinium HumanMethylation 27 K array, two publications [6, 33] have proposed splitting the CIMP-N subtype into two groups, one of which is enriched for distal tumours and TP53 mutations, and the other enriched for rectal tumours with a low frequency of mutations. Our hierarchical clustering dendrogram did not support the division of the CIMP-N subgroup into two groups, which might reflect the use of different clustering techniques or probe sets. Morever, a recent publication performed clustering based on 10,000 CpG probes also identified only three tumour subtypes [34].

An unexpected finding during our analysis was that a small number of tumours classified as CIMP-H had fewer hypermethylated genes than some CIMP-L tumours, and a small subset of CIMP-N tumours had a higher number of hypermethylated genes than some CIMP-L tumours. Thus, there is no distinct boundary in the number of hypermethylated genes between the CIMP subtypes, despite a difference in the average number of hypermethylated genes between the subtypes. The lack of distinct boundaries between CIMP groups might be explained by a variable number of stochastic hypermethylation events that contribute to the overall frequency of hypermethylated genes in each tumour.

The high density of the 450 K arrays enabled us to use the Wilcoxon Signed Rank test to interrogate methylation at all CpGs in each island and island shore. The benefit of this method is selection for genes with the greatest changes in methylation, and is an improvement on previous methods that identified hypermethylated genes on the basis of one or two differentially methylated CpGs. We identified over 2000 genes significantly differentially methylated between tumours and matched normal tissue. This is comparable to a previous study that mapped differential methylation to 1465 RefSeq genes [31]. Many of the genes observed to be differentially methylated have been identified previously, including *GATA4*/*5* [35], *SFRP2* [29], and the previously proposed serum and stool CRC marker genes *EYA4* [36] and *TFPI2* [37]. *EYA4* and *TFPI2*, along with *TLX1*, were the three most frequently hypermethylated genes in our dataset. Although, to the best of our knowledge, *TLX1* hypermethylation has not been previously associated with CRC, one study showed it is methylated in a high frequency of early stage breast cancers [38]. Activation of *TLX1* through either chromosomal relocation [39] or promoter CpG island demethylation [40] is associated with T-cell acute leukaemia. Notably, we observed multiple members of the *LHX*, *LMX*, *NKX*, *PAX* and *TBX* families of transcription factors were hypermethylated in all CIMP-H tumours. These transcription factor families have roles in development, spatial patterning and tissue homeostasis, and the aberrant silencing or expression of these genes has been associated with tumour growth kinetics and malignancy potential. *PAX* genes are widely expressed and associated with maintaining tissue homeostasis or wound repair and may play a role in maintaining progenitor cell pluripotency. Loss of expression and hypermethylation of *PAX* genes in cancer was recently reviewed in [41].

Mutations observed in tumours are commonly classified as driver or passenger events, the former being few in number, high in frequency and common to multiple tumour types, while the latter occur in many different genes and appear sporadically. This mutation landscape of tumours has been described as comprising ‘mountains’ (rare genes that are mutated frequently) and ‘hills’ (the many genes that are mutated rarely) [42]. Hypermethylation of genes also resembled this model, with many hypermethylated in a small number of tumours, and a smaller set of genes hypermethylated in the majority. When we scored only CIMP-H tumours the number of ‘mountains’ was much higher than observed in CIMP-N tumours. Strikingly, 132 genes are hypermethylated in 100% of CIMP-H tumours. These were highly enriched for roles associated with segment specification, morphogenesis, and development.

To explain the hypermethylation of 132 genes across all of our CIMP-H tumours we consider three potential models. The first model relies on natural selection, following disruption of the strict controls maintaining normal epigenome homeostasis, to shape all tumours towards a similar pattern (convergent evolution). Deletion of transcription factor binding sites leads to normally unmethylated CpG islands becoming progressively more methylated, thus the accumulation of methylation might represent a marker of dysregulated pathways in which transcription factor binding is no longer occurring [43]. The second model involves mutation of an upstream factor, e.g. transcription factor or chromatin remodeler, causing a specific set of genes to become hypermethylated (the “instructive model” [44, 45]). A study of gliomas, which also display the CpG island methylator phenotype (G-CIMP), demonstrated that the introduction of a single mutation in *IDH1* into primary human astrocytes rearranges the epigenome to match G-CIMP tumours [46]. This demonstrates that, at least in some tumour types, a single mutation can reproducibly induce genome-wide changes in methylation, however no single mutation that is sufficient to cause CIMP in CRC has yet been identified. Tahara et al., analysed CRC mutations and found the chromodomain genes *CHD7/8* frequently had non-silent mutations in CIMP-H tumours [47]. Further, the authors showed that genes previously identified as differentially methylated in CRC are frequently bound by CHD7. The activating *BRAF* V600E mutation is tightly correlated with the CIMP-H subtype [4]. *BRAF* activity has recently been reported to increase activity of the MAFG protein through the RAS-RAF pathway, leading to the recruitment of a repressor complex that facilitates promoter hypermethylation [48]. However, not all CIMP-H tumours carry this mutation, suggesting either MAFG activity is increased via a different mutation or an alternative mechanism of hypermethylation exists in CIMP-H tumours.

In the third model, CIMP-H tumours reflect the epigenetic state of the tumour-initiating cell. Tumour DNA methylation profiles have previously been shown to reflect their tissue of origin [49]. A plausible explanation for the drastically different epigenetic profiles of CIMP-H compared to CIMP-N tumours is that the tumour-initiating cell was in a different developmental state (e.g. progenitor compared to terminally differentiated). The developmental state of tumour-initiating cells has been shown to influence the characteristics of tumours [50]. Chow et al. used a single event, activation of the Sonic HedgeHog pathway, to transform both Neural Stem Cells (NSCs) and Neural Progenitor Cells (NPCs) into tumour-initiating cells. The tumours derived from NSCs and NPCs displayed different molecular characteristics, demonstrating the association between differentiation stage (or epigenetic state) of a tumour-initiating cell and the tumour subtype. The epigenetic state or differentiation stage of a cell might influence a resulting tumour through the pattern of histone modifications or the complement of transcription factors being expressed. We observed 89% of the genes hypermethyalated in all CIMP-H tumours were PcG targets (data not shown). In non-malignant cells, H3K27me3 (the repressive histone modification laid down by PcG proteins) provides a transient repression of transcription factors that, when activated, cause differentiation [51, 52]. This suggests tumours derived from cells at different stages of differentiation would acquire different hypermethylation profiles, given the established relationship between H3K27me3 and DNA methylation [13].

## Conclusion

In summary, the data presented here show that against a variable background of sporadic hypermethylation occurring in all CRC samples studied, a core set of genes are hypermethylated in CIMP-H tumours at a very high frequency. These core hypermethylation events may reflect selection pressures on the cell, the upstream disruption of critical cancer pathways, or, alternatively, highlight a distinct cell of origin for CIMP-H tumours.

## Additional file

Additional file 1: Table S1. 258 genes hypermethylated in more than 90% of all CIMP-H tumours. Two-hundred fifty eight CpG islands are hypermethylated in more than 90% of CIMP-H tumours. CpG islands hypermethylated in 100% of CIMP-H tumours are highlighted in bold. (XLSX 49 kb)

## Abbreviations

CIMP: CpG island methylator phenotype
CIMP-H/L/N: High/low/negative
CRC: Colorectal cancer
PcG: Polycomb group
MSI: Microsatellite instability
SAM: Significance analysis of microarrays
TSS: Transcriptional start site
